# Effect of Particle Size on Physical Properties, Dissolution, In Vitro Antioxidant Activity, and In Vivo Hepatoprotective Properties of *Tetrastigma hemsleyanum* Diels et Gilg Powders

**DOI:** 10.3390/pharmaceutics16111352

**Published:** 2024-10-23

**Authors:** Zhiwen Zhang, Yun Chen, Shaoxian Wang, Zheren Tong, Fujia Luan, Binghong Jiang, Faxiang Pu, Zhangfu Xie, Ping Wang, Zijin Xu

**Affiliations:** 1 Department of Pharmacy, Jiangxi Medical College, Shangrao 334000, China; 15297958867@163.com (Z.Z.); luanfj123321@163.com (F.L.); 13185879932@163.com (B.J.); 2 Department of Pharmacy, Jinhua Vocational and Technical University, Jinhua 321000, China; tiancy3348@163.com; 3 College of Pharmaceutical Sciences, Zhejiang University of Technology, Hangzhou 310014, China; wangshaoxianmail@163.com (S.W.); 2112007040@zjut.edu.cn (Z.T.); 4 Zhejiang Suichang Liming Pharmaceutical Co., Ltd., Suichang 323300, China; pfx888@163.com (F.P.); xiezhangfu1999@sina.com (Z.X.)

**Keywords:** *Tetrastigma hemsleyanum* Diels et Gilg, powders, particle size, physical properties, dissolution, hepatoprotective

## Abstract

**Objective:** The aim of this study was to analyze the effects of different particle sizes of *Tetrastigma hemsleyanum* Diels et Gilg (TDG) powders on physical properties, dissolution, in vitro antioxidant activity, and in vivo hepatoprotective properties. **Methods:** The particle size of TDG coarse powders (TDG-CP), TDG fine powders (TDG-FP), and TDG micro powders (TDG-MP) were measured by a laser particle size analyzer. The physical properties were measured according to the latest version of the Chinese Pharmacopoeia (Committee Chinese Pharmacopoeia 2020). The content of the total flavonoids, total polysaccharides, kaempferol-3-O-rutinoside, and rutin of TDG powders were determined using the NaNO_2_-Al (NO_3_)_3_ colorimetric method, the sulphate-phenol colorimetric method, and HPLC, respectively. In vitro dissolution and antioxidant activity were determined by the paddle method in phosphate buffer (pH 6.8) and the DPPH radical scavenging method, respectively. In addition, the liver tissue pathology was evaluated by hematoxylin and eosin staining (H&E), and the AST and ALT activities were measured by automatic biochemical analyzer. The superoxide dismutase (SOD), catalase (CAT), and glutathione (GSH) activities were measured by using commercial analysis kits. **Results:** As the particle size decreases, the fluidity of TDG powders decreased and the porosity increased. In addition, there were no significant differences in physical properties between low temperature pulverized powders and room temperature pulverized powders. The final dissolution rates of the four bioactive ingredients in TDG-MP were found to be 85.06%, 85.61%, 83.88%, and 83.26%, respectively, whereas in TDG-CP, the dissolution rates were significantly lower at 18.79%, 17.96%, 22.46%, and 24.35%. The EC_50_ values of TDG-CP, TDG-FP, and TDG-MP on DPPH scavenging activity were 0.82, 0.31, and 0.10 mg/mL, respectively. The AST and ALT activities of the TDG-FP group and the TDG-MP group were significantly decreased and the SOD, CAT, and GSH activities were significantly increased when compared with that of the model group. The inflammatory cell infiltration and vacuolar degeneration of liver cells in the TDG-FP group and the TDG-MP group were significantly improved. **Conclusions:** The particle size of TDG powders had a significant effect on the physical properties and in vivo bioactivity. TDG pulverized to a fine particle size or smaller is a promising approach for clinical applications with improved physicochemical and biological properties.

## 1. Introduction

*Tetrastigma hemsleyanum* Diels et Gilg (TDG), a perennial plant species belonging to the genus *Tetrastigma Planch*, family *Vitaceae*, is widely distributed in the provinces of Zhejiang, Jiangxi, Fujian, Guangxi, and Yunnan provinces in China [1]. According to traditional Chinese medicine theory, TDG is believed to have the effect of clearing heat and detoxifying, promoting blood circulation, and dispelling wind and eliminating phlegm [2]. Modern pharmacological studies show that TDG has many pharmacological activities and is anti-tumor, antiviral, anti-inflammatory, hepatoprotective, antipyretic, analgesic, immune enhancing, etc. [3,4,5]. Of those, the beneficial hepatoprotective effects of TDG have been confirmed by a number of studies [6]. Nearly 200 chemical components have been found in TDG, mainly consisting of flavonoids, polysaccharides, phenolic acids and their derivatives, triterpenoids, organic acids, fatty acids, etc., among which flavonoids of TDG have received the most attention for their health-promoting properties [6,7,8]. More than 100 kinds of flavonoids have been found in TDG, among which flavonoid glycosides account for the largest proportion [6].

It is commonly understood that the preparation of decoctions using water is a widely utilized therapeutic modality in herbal medicine [9]. However, it is intriguing that the detailed methods of TDG usage described in ancient medical literature include “water decoction” and “mashed into pulp with cold water.” This suggests that certain active ingredients are temperature sensitive and may be degraded in the process of water decoction. TDG is not recorded in the current version of the Chinese Pharmacopoeia, but fresh TDG, TDG decoction slices, and TDG powders are registered in the local standards of different provinces in China. The fresh TDG should not be stored for a long time, and the active ingredients may be destroyed in TDG decoction slices. Therefore, the preparation of TDG into powders after drying has become the most widely clinically accepted, and a number of commercial products have been listed. It is a well-acknowledged viewpoint that the active ingredients are the potential bioactive material basis of medicinal herbs [10]. The previous work by our team showed that kaempferol-3-O-rutinoside and rutin are the most important active ingredients in TDG, which can be used for the quality control of TDG [2]. Furthermore, the powders of TDG with different particle size can be obtained by crushing by different processes. Due to the lighter degree of plant tissue breakage and smaller specific surface area, the powders with a large particle size limit the dissolution rate of the active ingredients in the gastrointestinal tract after oral administration [11,12]. In contrast, the active ingredients in the smaller particle size powders are more easily released, and this improves their bioavailability and efficacy [13,14,15]. Therefore, particle size is determined to be a critical quality attribute (CQA) of TDG powders. In a prior investigation, the particle size distribution of commercially available TDG powders was examined, revealing that the majority of samples exhibited particle sizes between 100 μm and 400 μm. Furthermore, the particle size resulting from folk application of basic grinding techniques approximated that of the coarse powder as defined in the Chinese Pharmacopoeia (unpublished data). Unfortunately, there is no limit standard for the particle size of TDG powders that can be followed in the current version of local standards, and this may lead to a difference in clinical efficacy.

In this study, TDG powders with different particle sizes were prepared by different process procedures, and the physical properties, including particle size, angle of repose, angle of slip, bulk density, and tap density, were analyzed. Meanwhile, the contents of total flavonoids, total polysaccharides, kaempferol-3-O-rutinoside, and rutin in different particle sizes of TDG powders were determined. In addition, the in vitro dissolution and antioxidant activity of TDG powders with different particle sizes were determined by the paddle method in phosphate buffer (pH 6.8) and the DPPH radical scavenging method, respectively. Finally, CCl_4_-induced liver injury model mice were used to evaluate the hepatoprotective quality of TDG with different particle sizes.

## 2. Materials and Methods

### 2.1. Plant Materials

The TDG was purchased from Shangrao Hongri Agricultural Development Co., Ltd. (HuaiYushan Country, Yushan Town, Shangrao City, Jiangxi Province, China) and the botanical identification was made according to Zhejiang Traditional Chinese Medicine Processing Standards (2015 edition). One certificate specimen (No. TDG 20230105) is stored in the herbarium of Jiangxi Medical College. Fresh TDG was freeze-dried and stored at room temperature for further use.

### 2.2. Chemical Reagents

Kaempferol-3-O-rutinoside (purity ≥98%) was purchased from the Chengdu Desite Biotechnology Co., Ltd. (Chengdu, Sichuang, China). Rutin (purity ≥98%), glucose, silibinin, and hematoxylin-eosin (H&E) dyeing solution were purchased from Shanghai Yuanye Biotechnology Co., Ltd. (Shanghai, China). An AST kit, ALT kit, CAT kit, GSH kit, and SOD kit were purchased from Nanjing Jiancheng Bioengineering Institute (Nanjing, Jiangsu, China). A BCA protein concentration assay kit was purchased from Shanghai Beyotime Biotechnology Co., Ltd. (Shanghai, China). DPPH acquired by Ruji Biotechnology Co., Ltd. (Shanghai, China). CCl_4_ was purchased from Shanghai Macklin Biochemical Technology Co., Ltd. (Shanghai, China). The acetonitrile was chromatographic grade, and other reagents were analytically pure.

### 2.3. Preparation of Samples with Different Particle Sizes

The TDG decoction slices were prepared according to the general preparation procedure in the Chinese Pharmacopoeia. Briefly, fresh TDG was washed with clear water and drained, cut into 3–5 mm slices, and freeze dried [16]. In order to obtain TDG powders with different particle sizes, TDG decoction slices were crushed by a mortar, a multifunctional pulverizer, and a fully automatic rapid grinding instrument. Detailed preparation methods are shown in Table 1.

### 2.4. Determination of the Physical Properties of Samples of Different Particle Sizes

#### 2.4.1. Measurement of the Particle Size Distribution

Using distilled water as a dispersing medium, the particle size distribution of TDG powders with different particle sizes was determined by the wet method with a Malvern laser particle size measuring instrument (Malvern M3000, Malvern Instrument Ltd., Malvern, UK).

#### 2.4.2. Measurement of the Angle of Repose

The angle of repose of TDG powders with different particle sizes was measured by the fixed funnel method [17]. Three funnels were fixed in series on an iron rack, and a glass plate was placed at the lower end of the funnel (the lower end of the funnel was 3 cm away from the surface of the glass plate). The TDG powders with different particle sizes were added to the funnel and made to flow out of the funnel slowly and evenly until the cone on the glass plate contacted the lower end of the funnel. The height (*h*) and diameter (*R*) of the cone formed by the sample were measured. According to formula 1, the angle of repose (*θ*) of the powders with different particle sizes was calculated as

(1) $$tan\;\theta =\frac{2h}{R},$$

where *h* is the height of the cone and *R* is the diameter of the cone.

#### 2.4.3. Measurement of the Slip Angle

The TDG powders with different particle sizes were laid on the glass panel, and then the panel was slowly lifted from one side until 90% of the sample moved. At this time, the angle between the plate and the horizontal plane is the slip angle.

#### 2.4.4. Measurement of Bulk Density

The measuring cylinder with a scale of 50 mL was taken and weighed accurately; its mass was *M*_1_ and the powders of different particle sizes were added slowly into the measuring cylinder until it was exactly flush with the scale (you could use the scraper to carefully smooth the surface being careful not to compact the powders), the powders attached to the outer wall of the sample receiving cup were removed and the total mass was weighed accurately as *M*_2_. According to Formula (2), the bulk density of the TDG powders with different particle sizes was calculated [18] as

(2) $$\rho =\frac{M_{2}-M_{1}}{50}\times 100\%,$$

where, *M*_1_ is the mass of the empty cylinder and *M*_2_ is the total mass of the cylinder and powders.

#### 2.4.5. Measurement of the Tap Density

A 15 mL metal container with a lid was taken and weighed accurately; its mass was *M*_1_. TDG powders with different particle sizes were added into the metal container, and the metal container was subjected to 200 vibrations at a frequency ranging from 50 to 60 vibrations per minute. The operation was repeated until the sample overflowed, and the surface was scraped carefully with a scraper to remove the powders attached to the outer wall of the sample receiving cup. The total mass was weighed accurately as *M*_2_. According to Formula (3), the tap density of the TDG powders with different particle sizes was calculated [19] as

(3) $$\rho =\frac{M_{2}-M_{1}}{15}\times 100\%,$$

where *M*_1_ is the mass of the empty container, and *M*_2_ is the total mass of the container and powders.

#### 2.4.6. Identification of Microscopic Features

TDG powders with different particle sizes were placed on a slide, permeated with chloral hydrate, fixed with glycerin, and then observed under a 20-objective microscope (Olympus Microscope System CKX53; Olympus, Tokyo, Japan). The characteristic structures were photographed and compared.

### 2.5. Determination of the Bioactive Ingredients

#### 2.5.1. Determination of Total Flavonoid Content

Two g of the TDG powders were weighed accurately into a beaker, then 40 mL of 80% methanol solution was added, and the weight was recorded. After reflux extraction for 1 h, the solution was cooled to room temperature, 80% methanol was added to make up for the weight loss, and the test solution was obtained. The content of total flavonoids in TDG powders was determined by the NaNO_2_-Al(NO_3_)_3_ colorimetric method [20]. Briefly, 1 mL of the sample solution with a final concentration of 5 mg/mL (calculated based on the concentration of raw medicinal materials) was placed in a 20 mL stoppered test tube, water was added to make it up to 6 mL, 1 mL 5% sodium nitrite solution was added, the solution was shaken well and left to stand for 6 min, then 1 mL 10% aluminum nitrate solution was added precisely; similarly, the solution was shaken well and left to stand for 6 min. Finally, 10 mL of 4% sodium hydroxide solution was added precisely, the solution was shaken well and left to stand for 15 min, and the absorbance value was determined at 500 nm by TU-1800PC UV-visible spectrophotometry (TU-1800PC, Puxi General Instruments Ltd., Beijing, China).

#### 2.5.2. Determination of Total Polysaccharide Content

Two g of the TDG powders were weighed accurately into a beaker, 40 mL water was added, and the weight was recorded. The solution was heated in a boiling water bath for 1 h and then cooled to room temperature; water was added to make up the weight loss. Centrifugation took place at 4000 rpm for 20 min, the supernatant was taken, and 4 times the volume of anhydrous ethanol was added to it for precipitation. The solution was kept overnight at 4 °C and then centrifuged at 4000 rpm for 20 min. Finally, the precipitate was taken and dissolved in 20 mL water and a sample solution was obtained. The total polysaccharide content with the TDG powders was determined by sulfuric acid-phenol colorimetry [21]. Briefly, 0.5 mL of the sample solution was accurately measured and placed in a 10 mL stoppered test tube. Then the solution was supplemented with water to 1 mL and shaken well. A solution of 1.2 mL 6% phenol and 5 mL concentrated sulfuric acid were added in the solution, then the mixed solution was shaken well, placed in a 40 °C water bath for 10 min, removed, and cooled at room temperature; the absorbance was determined as 490 nm by UV-visible spectrophotometry (TU-1800PC, Puxi General Instruments Ltd., Beijing, China).

#### 2.5.3. Determination of the Kaempferol-3-O-Rutinoside and Rutin Content

The determination of the kaempferol-3-O-rutinoside and rutin content was conducted according to the method reported by our team previously [2]. The sample solution was prepared by the same extraction method as that used in the determination of the total flavonoids. The sample solution with a final concentration of 0.2 g/mL (calculated based on the concentration of raw medicinal materials) was filtered by a 0.22 μm filter membrane. Then, the filtrate was analyzed by HPLC (LC-20A, SHIMADZU Instruments ltd., Kyoto, Japan). The chromatographic conditions were as follows: Shim-pack VP-ODS-C18 column (4.6 mm × 250 mm, 5 μm); mobile phase A: acetonitrile; mobile phase B: 0.1% acetic acid-aqueous solution; flow rate: 0.5 mL/min; column temperature: 30 °C; detection wavelength: 300 nm; gradient elution procedures are shown in Table 2.

### 2.6. Dissolution In Vitro

The third method (small cup method) stipulated in the determination method of dissolution and release in the Chinese Pharmacopoeia (2020 Edition Four, General Rule 0931) was referenced to determine dissolution [22]. Ten g of TDG powders with different particle sizes were added to 150 mL of a phosphate buffer with pH 6.8, the temperature was set at 37± 0.5 C, and the rotating speed of the stirring paddle was 75 rpm. A 5 mL sample was taken at 15, 30, 45, and 60 min for detection, and the same volume of dissolution medium was added immediately after sampling.

### 2.7. Antioxidant Activities

The antioxidant activities of the TDG powders with different particle sizes were assessed by DPPH assay [23]. The sample solution was prepared by the same extraction method as that used in the determination of total flavonoids, and the final concentration was adjusted to 25 mg/mL (calculated based on the concentration of raw medicinal materials). Further, the sample solution was diluted to a series of concentrations as needed for the actual assay with 80% methanol for further use. The standard DPPH solution was diluted to 5, 10, 20, 25, 30, and 40 μg/mL, and the absorbance value was measured at 517 nm by UV-visible spectrophotometry (TU-1800PC, Puxi General Instruments ltd., Beijing, China) with 80% methanol as blank. A standard curve was prepared by determining the decrease in absorbance of the DPPH radical solution.

Into a brown stoppered test tube, 5 mL of DPPH standard solution (20 μg/mL) and 0.5 mL of TDG sample solution ranging from 0.05 to 2.5 μg/mL were added. Under the condition of avoiding light, the tube was placed in a water bath for 30 min at 30 °C. The absorbance value (*A* value) was measured at 517 nm by UV-visible spectrophotometry (TU-1800PC, Puxi General Instruments Ltd., Beijing, China) with 80% methanol as blank. The DPPH radical scavenging rate was calculated according to Formula (4):

(4) $$DPPH\%=\frac{A_{0}-A}{A_{0}}\times 100\%,$$

where *A*_0_ is the absorbance value of the blank sample and *A* is the absorbance value of the sample.

### 2.8. Liver Protection Performance

#### 2.8.1. Animals and Groups

Male ICR mice (weight range of 18~22 g) were purchased from Shanghai Jihui Experimental Animal Breeding Co., Ltd. (Shanghai, China). (experimental animal license number: SCXK (Su)-2019-0008). Among the mice, 48 were divided randomly into 6 groups (in every group *n* = 8), including a control group, model groups, a positive drug group (silibinin), a TDG coarse powders group (TDG-CP), a TDG fine powder group (TDG-FP), and a TDG micro powders group (TDG-MP). TDG powders with different particle sizes and silibinin were suspended in a 0.5% sodium carboxymethyl cellulose (CMC-Na) solution. After 3 days of adaptive feeding in the SPF laboratory, the mice in each group were given the drug on the 4th day and on 7 consecutive days. The mice in the TDG-CP group, the TDG-FP group, and the TDG-MP group were given TDG powders with different particle sizes by intragastric administration at a dose of 3 g/kg/day (calculated based on the concentration of raw medicinal materials). The mice in the positive drug group were given silibinin by intragastric administration at a dose of 200 mg/kg/day, and the administration volume of each group was 0.2 mL/10 g. The mice in the control group and the model group were given 0.5% CMC-Na solution by intragastric administration. One hour after the last administration, the mice in the groups other than the control group were injected intraperitoneally with 0.2% CCl_4_ soybean oil solution, and the injection volume was 0.1 mL/10 g, while the mice in the control group were injected with the same volume of soybean oil [24]. After fasting for 16 h, 1.5 mL of orbital blood was taken from the mice, and then the mice were killed by cervical dislocation under deep anesthesia with isoflurane. A liver specimen was removed from the animal and immediately divided into two parts. One portion was frozen and stored at −80 °C for further analysis, and the other portion was fixed in 10% neutralized formalin for paraffin embedding and histopathological evaluation (H&E).

#### 2.8.2. Determination of AST and ALT Activities

The blood of the mice in each group was centrifuged twice at 3000 rpm for 10 min, and the supernatant was collected carefully. The serum AST and ALT activities were detected using an automatic biochemical analyzer (Hitachi 7020, Hitachi Ltd., Tokyo, Japan).

#### 2.8.3. Determination of SOD, CAT, and GSH Activities in the Liver

The livers of mice in each group were washed with normal saline, dried with filter paper, weighed, and photographed to record the surface morphology of the livers. In each group, 0.2 g of the remaining liver tissue from the mice was collected and homogenized in pre-cooled normal saline using an automatic rapid grinding instrument to prepare a 10% liver tissue homogenate. The homogenate was then centrifuged at 12,000 rpm for 10 min, and the resulting supernatant was carefully collected. The protein concentration in each sample was determined using a BCA protein quantitative test kit. Subsequently, the activities of SOD, CAT, and GSH in the liver tissue of mice in each group were determined using commercially available kits following the manufacturers’ instructions.

#### 2.8.4. Liver Histopathological Examination of the Mice

The histopathological examination of the liver involved the collection of liver lobules from a consistent location, fixing them in 10% neutral buffered formalin for 72 h, embedding them in paraffin, sectioning them to a thickness of 5 µm, staining them with hematoxylin and eosin, and observing them at 200× magnification using a microscope followed by photographic documentation [25].

### 2.9. Statistical Analysis

The mean ± standard deviation (± SD) was used to represent the data of each group. Statistical analysis was performed with one-way analysis of variance (ANOVA) using SPSS 22.0 software. The result of *p* < 0.05 was considered statistically significant.

## 3. Results

### 3.1. Physical Properties of Three Leaf Green Powders with Different Particle Sizes

The physical characteristics of the TDG powders with different particle sizes, such as particle size distribution, angle of repose, angle of slip, bulk density, and tap density, are presented in Table 3. The findings indicate that diverse particle sizes of TDG powders can be achieved through various grinding methods, including a Chinese medicine pulverizer and an automatic rapid grinding instrument. Following processing with the automatic rapid grinding instrument, the D_50_ of TDG-MP was measured at 16.2 μm, indicating the attainment of micro-powder levels. As the particle size decreases, the span of the size distribution of TDG powder progressively broadens. This phenomenon may be attributed to the grinding process, where insufficient ball milling could result in a larger particle size distribution span of the TDG powders. The angle of repose and the angle of slide for the powders exhibited an increase from 32.60° and 53.01° for TDG-FP to 29.25° and 40.11° for TDG-MP, with the smallest values observed for TDG-CP at 25.79° and 32.02°. Similarly, the bulk density and tap density of the powders showed an increase from 0.47 g/mL and 0.49 g/mL for TDG-FP to 0.70 g/mL and 1.01 g/mL for TDG-MP, while the coarse powders had the smallest values at 0.42 g/mL and 0.47 g/mL. In summary, as the particle size of the TDG powders decreases, fluidity decreases and porosity increases. Furthermore, there is no notable disparity in the physical properties between low-temperature powders and their normal temperature counterparts, aligning with the findings of particle size analysis.

### 3.2. Microscopic Characteristics of Different Particle Sizes of TDG Powders

**Figure 1:**
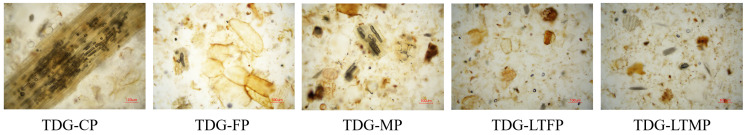
Microstructure of TDG powders with different particle sizes. Scale bar: 100 μm.

The microstructure of TDG powders with different particle sizes are illustrated in Figure 1. The findings indicate that TDG-CP exhibits typical root and stem characteristics resembling those found in plants, with relatively intact cell structures and distinct duct cells. TDG-FP displays clear cork cells, vessels, calcium oxalate crystals, and brown fragments, with minimal cell damage. Conversely, TDG-MP exhibits significant cell structure damage and smaller cell sizes, although cork cells, vessels, calcium oxalate crystals, brown fragments, and other features are still discernible. Additionally, the analysis revealed that there is minimal variation in cell structure between the low-temperature treatment and the normal temperature treatment, suggesting that the normal temperature treatment does not significantly impact the cell structure of the powders.

### 3.3. Results of Bioactive Ingredients Content

UV-visible spectrophotometry and HPLC were used for the determination of the bioactive ingredient content of the TDG powders. The analysis of the bioactive ingredient content of the TDG powders with different particle sizes revealed that the highest concentration of total flavonoids was 23.63 ± 1.57 mg/g, while the peak concentration of total polysaccharides reached 31.1 ± 1.16 mg/g. Additionally, the maximum concentration of kaempferol-3-O-rutinoside was determined to be 75.60 ± 2.41 μg/g, and the highest concentration of rutin was found to be 56.20 ± 2.02 μg/g. These quantified values were subsequently employed as the bioactive ingredient content in the TDG powders for the purpose of dissolution calculations.

### 3.4. Effects of TDG Powders with Different Particle Sizes on In Vitro Dissolution

**Figure 2:**
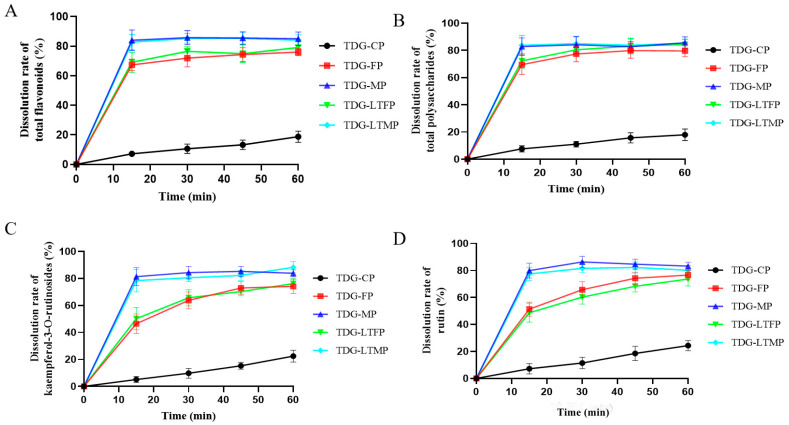
Effects of TDG powders with different particle sizes on in vitro dissolution. (**A**) Total flavonoids; (**B**) total polysaccharides; (**C**) kaempferol-3-O-rutinoside; (**D**) rutin. Values are expressed as mean ± SD (*n* = 6).

The outcomes of in vitro dissolution of the bioactive ingredients of the TDG powders with different particle sizes are illustrated in Figure 2. The final dissolution rates at 60 min of total flavonoids, total polysaccharides, kaempferol-3-O-rutin, and rutin in TDG-CP are 18.79%, 17.96%, 22.46%, and 24.35%, respectively, whereas those in TDG-FP are 76.04%, 79.60%, 74.19%, and 76.64%, respectively. TDG-MP exhibits notably higher dissolution rates, with the dissolution rates at 60 min for the 4 bioactive ingredients being 85.06%, 85.61%, 83.88%, and 83.26%, respectively. It can be seen that the dissolution rate and final cumulative dissolution amount of TDG-MP are significantly higher than that of TDG-CP.

### 3.5. Effects of TDG Powders with Different Particle Sizes on Antioxidant Activities

**Figure 3:**
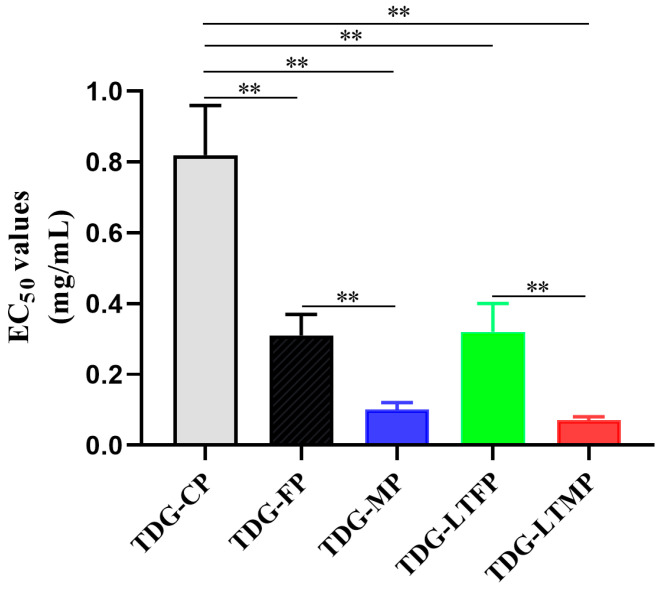
Effects of TDG powders with different particle sizes on antioxidant activities. Values are expressed as mean ± SD (*n* = 4). ** *p* < 0.01.

The EC_50_ values of the DPPH free radical scavenging ability of TDG powders with different particle sizes are shown in Figure 3. The results showed that TDG powders with different particle sizes showed different levels of antioxidant activity. Specifically, the EC_50_ values of the DPPH free radical scavenging of TDG-CP, TDG-FP, and TDG-MP were 0.82 mg/mL, 0.31 mg/mL, and 0.10 mg/mL, respectively. There were significant differences among the three groups. These results suggest that smaller particle sizes correspond to the increased antioxidant efficacy of the TDG powders. The temperature of the crushing process did not have a significant impact on the antioxidant activity of the TDG powder, which is consistent with the findings related to particle size and dissolution.

### 3.6. Effects of TDG Powders with Different Particle Sizes on Serum AST and ALT Activities

**Figure 4:**
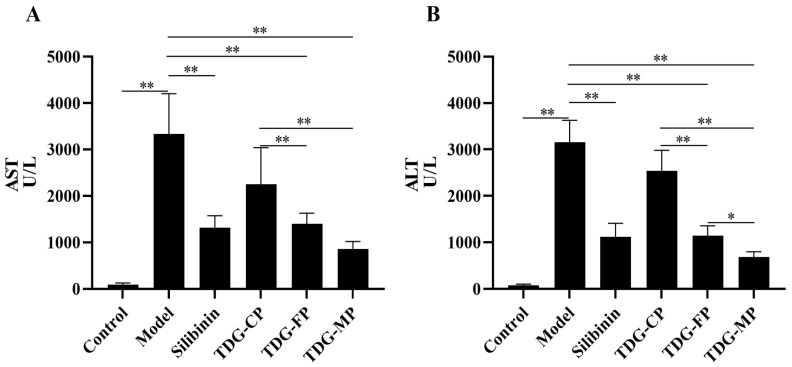
Effects of TDG powders with different particle sizes on serum AST (**A**) and ALT (**B**) activities. Values are expressed as mean ± SD (*n* = 8). ** *p* < 0.01, * *p* < 0.05.

The effects of TDG powders with different particle sizes on serum AST and ALT activities are illustrated in Figure 4. In comparison to the control group, the model group exhibited a significant increase in serum AST and ALT activities, suggesting successful establishment of an acute liver injury model in mice induced by CCl_4_. Conversely, both the silibinin group and the groups treated with TDG powders with different particle sizes demonstrated a decrease in serum AST and ALT activities when compared to the model group. There was no statistically significant difference observed between the TDG-CP group and the model group; however, the silibinin group, TDG-FP group, and TDG-MP group exhibited significant differences when compared with the model group. Furthermore, a significant difference in ALT activity was observed among the three groups of TDG powders with different particle sizes. However, no significant difference in AST activity was detected between the TDG-FP group and the TDG-MP group. These findings suggest that the hepatoprotective effect of the TDG powders in vivo becomes more pronounced with decreasing particle size, aligning with the results of the dissolution testing in vitro. Notably, TDG-MP demonstrated a more pronounced protective effect against CCl_4_-induced acute liver injury in mice when compared to TDG-CP and TDG-FP.

### 3.7. Effects of TDG Powders with Different Particle Sizes on Liver Morphology

**Figure 5:**
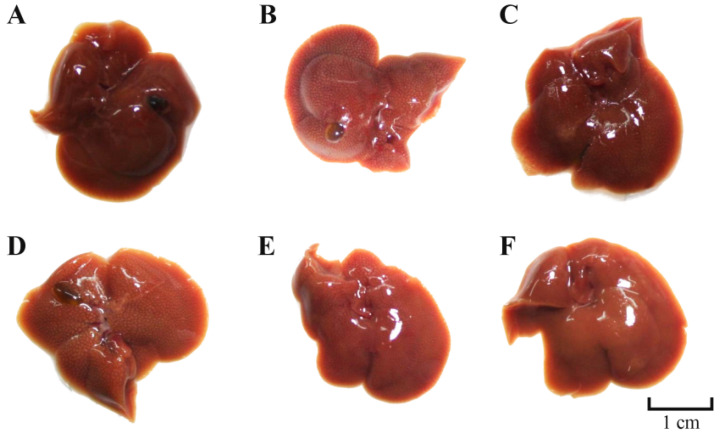
Effects of TDG powders with different particle sizes on liver morphology. (**A**) Control group; (**B**) model group; (**C**) silibinin group; (**D**) TDG-CP group; (**E**) TDG-FP group; (**F**) TDG-MP group.

As illustrated in Figure 5, the livers of mice in the control group exhibited a reddish-brown color, soft texture, smooth surface, and well-defined edges, with no apparent damage observed on the liver surface. In contrast, the livers of mice in the model group displayed signs of swelling, increased hardness, and noticeable necrotic lesions on the surface. A marked reduction in liver damage was observed in the silibinin group, the TDG-FP group, and the TDG-MP group. When compared to the model group, the liver color of mice in each group more closely resembles that of the control group, the liver texture is softer than that of the model group, and the presence of necrosis spots on the liver surface is significantly reduced, particularly in the TDG-MP group. These findings suggest that TDG-MP exhibits a more pronounced protective effect against CCl_4_-induced acute liver injury in mice compared to TDG-CP and TDG-FP, potentially due to its active ingredients being more readily dissolved and absorbed, resulting in superior efficacy in vivo.

### 3.8. Effects of TDG Powders with Different Particle Sizes on Liver SOD, CAT, and GSH Activities

**Figure 6:**
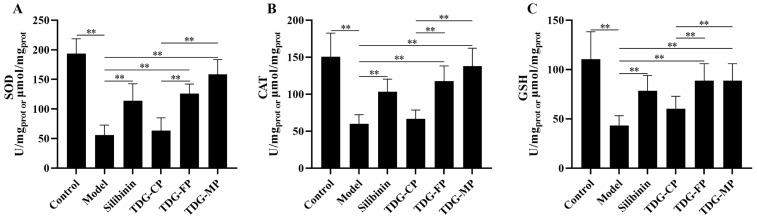
Effects of TDG powders with different particle sizes on liver SOD (**A**), CAT (**B**), and GSH (**C**) activities. Values are expressed as mean ± SD (*n* = 8). ** *p* < 0.01.

The effects of TDG powders with different particle sizes on the activities of SOD, CAT, and GSH is illustrated in Figure 6. Compared to the control group, the activities of SOD, CAT, and GSH in the livers of mice treated with CCl_4_ are significantly decreased, indicating the successful establishment of an acute liver injury model induced by CCl_4_ in mice. Compared to the model group, the activities of SOD, CAT, and GSH in the silibinin, TDG-FP, and TDG-MP groups showed differing degrees of elevation, while no significant difference was observed between the TDG-CP group and the model group. Furthermore, notable differences were observed in the activities of SOD, CAT, and GSH between the TDG-FP and TDG-MF groups compared to the TDG-CP group. However, no significant differences were detected between the TDG-FP and TDG-MP groups.

### 3.9. Effects of TDG Powders with Different Particle Sizes on Liver Histopathology

**Figure 7:**
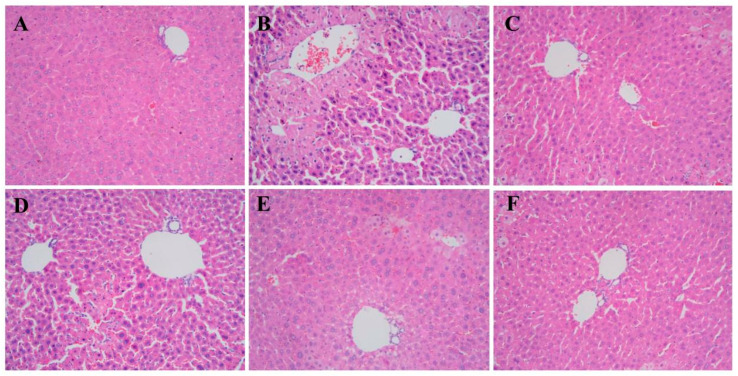
Effects of TDG powders with different particle sizes on liver histopathology (200×). (**A**) Control group; (**B**) model group; (**C**) silibinin group; (**D**) TDG-CP group; (**E**) TDG-FP group; (**F**) TDG-MP group.

The findings of liver histopathology are illustrated in Figure 7. In the control group, the hepatic tissue exhibits a well-defined and organized structure, with liver cell cords arranged in a radial pattern around the central vein, displaying normal nuclear size. In contrast, the liver tissue of the model group displays a disrupted structure, disordered arrangement of liver cell cords, spotty and flakelike necrosis in liver cells, infiltration of inflammatory cells, and vacuolar degeneration. The histopathological characteristics of mice in the silibinin group, the TDG-FP group, and the TDG-MP group show significant improvement, with hepatocyte necrosis, inflammatory cell infiltration, and vacuolar degeneration being notably absent in the TDG-MP group.

## 4. Discussion

There are a variety of dosage forms for traditional Chinese herbal medicinal products, such as decoctions, powders, pills, wines, concentrates, ointments, tinctures, syrups, suppositories, and more [26]. Powders occupy a pivotal position in traditional dosage form, and they can be available for oral and topical use [27]. The particle size of traditional Chinese medicine powders was identified as a potential critical quality attribute impacting physical properties, the solubility of bioactive ingredients, and therapeutic effect [28]. The present study first evaluated the variations in physical properties, the in vitro dissolution, antioxidant activity, and bioactive ingredients content of TDG powders with different particle sizes. On the basis of the various pulverizing scales, physical properties, including particle-size distributions, angle of repose *θ*, bulk density, and tap density were investigated. The findings indicate a notable alteration in the fluidity and compressibility of the powder as the particle size decreases. TDG-FP and TDG-MP exhibit inferior fluidity and compressibility in comparison to TDG-CP, a phenomenon attributed to the heightened specific surface area of the powder. As per the Noyes–Whitney equation, the specific surface area of the powder is directly related to the dissolution rate [29]. During in vitro dissolution, the reduction in particle size of TDG powders enhances the surface area available for interaction with the dissolution medium, facilitating the rapid dispersion of total flavonoids, total polysaccharides, kaempferol-3-O-rutin, and rutin in TDG. These ingredients have been previously recognized as the principal bioactive ingredients of TDG. Moreover, TDG-FP and TDG-FP exhibit a higher degree of fragmentation under microscopic observation, attributed to their increased crushing strength, thereby reducing the diffusion resistance of bioactive ingredients, which provides favorable conditions for the dissolution of bioactive ingredients [11]. The study found that the antioxidant activity of TDG-MP was superior to that of TDG-FP, with TDG-CP exhibiting the lowest activity, consistent with in vitro dissolution results. The rapid dissolution of bioactive ingredients has been shown to enhance antioxidant activity in vitro, as supported by previous research. While previous studies have suggested that heat generated during grinding may lead to the degradation of active ingredients, our study found no significant difference in dissolution and antioxidant activity between low-temperature and room-temperature grinding processes, suggesting that TDG can be crushed at room temperature [30,31].

This study assessed the potential hepatoprotective properties of TDG-CP, TDG-FP, and TDG-MF against acute liver injury induced by CCl_4_. The examination of AST, ALT, SOD, CAT, GSH activities, liver morphology, and liver histology revealed that TDG-MP exhibited the most pronounced hepatoprotective properties, whereas TDG-CP did not demonstrate a significant difference compared to the model group. The AST and ALT activities serve as crucial markers for assessing liver function, with elevated levels typically indicating liver cell injury [32]. The results of this study revealed that the AST and ALT activities in the TDG-MP group were significantly reduced compared to those in the model group, suggesting that TDG-MP administration effectively mitigated liver cell damage induced by CCl_4_. SOD, CAT, and GSH constitute critical elements of the antioxidant enzyme system, and reductions in their activities are frequently linked to the exacerbation of oxidative stress [33,34]. Oxidative stress is implicated in the pathophysiology of acute liver injury, a relationship that has been substantiated by numerous studies. Consequently, SOD, CAT, and GSH have been employed as biomarkers for liver injury in various research investigations [35,36]. Previous studies have demonstrated that TDG reduces MDA levels and restores the activities of liver antioxidant enzymes, including SOD, GSH-Px, and CAT. These findings suggest its potential role in mitigating oxidative stress, a conclusion further corroborated by the present study [37]. The activities of SOD, CAT, and GSH in the TDG-MP group exhibited a notable increase compared to those in the model group, suggesting that TDG-MP has the potential to bolster the antioxidant defense system and mitigate liver cell injury induced by oxidative stress following oral administration. The enhanced efficacy of TDG-MP may be attributed to its reduced particle size and increased surface area, which promote the efficient release of bioactive ingredients from the powder and their rapid absorption in the body, ultimately enhancing bioavailability and resulting in heightened liver protection. This study expands upon the existing research by demonstrating the protective effects of TDG on liver injury. Furthermore, it is the first to compare the impact of TDG powders with different particle sizes on liver injury and offer a comprehensive molecular mechanism explanation. These findings offer valuable insights for the advancement and implementation of TDG preparations in clinical settings.

## 5. Conclusions

This study comprehensively assessed TDG powders with different particle sizes in terms of physical properties, in vitro dissolution, antioxidant activity, and hepatoprotective properties in vivo. The findings indicate that TDG micro powders with a smaller particle size outperformed coarse and fine powders across all evaluated parameters. Furthermore, no significant disparities were observed in the physical properties, in vitro dissolution, and antioxidant activity between TDG powders produced through normal and low temperature pulverization methods. This suggests that the room temperature pulverization method is suitable for producing TDG micro powders. This discovery offers a theoretical foundation for the expanded utilization of TDG, particularly in situations necessitating rapid dissolution and effective absorption, such as in the formulation of oral medications. Additionally, the outcomes offer practical assistance for the large-scale manufacturing of TDG, demonstrating that micronization is a viable strategy for enhancing the therapeutic effectiveness of TDG.

## Figures and Tables

**Table 1:** Preparation methods of TDG powders with different particle sizes.

TDG Powders	Crushing Methods
Coarse powders (TDG-CP)	The TDG decoction slices were mashed in a mortar and filtered through a 40-mesh sieve.
Fine powders (TDG-FP)	The TDG decoction slices were crushed with a Chinese medicine pulverizer for 1 min and filtered through a 120-mesh sieve.
Micro powders (TDG-MP)	First, the TDG decoction slices were crushed with a mortar, the powders were passed through a 120-mesh sieve, and then the powders were added to the fully automatic rapid grinding instrument (JX-2GL, Shanghai Jingxin Industrial Development Co., Ltd., Shanghai, China) with a grinding power of 60 Hz. They were ground a total of 3 times, with a grinding time of 30 s, and a grinding interval of 15 s.
Low temperature fine powders (TDG-LTFP)	The TDG decoction slices were crushed with a Chinese medicine pulverizer for 1 min after being soaked in liquid nitrogen for 10 min and then filtered through a 120-mesh sieve.
Low temperature micro powders (TDG-LTMP)	First, the TDG decoction slices were crushed with a mortar and filtered through a 120-mesh sieve, then the powders were added to the fully automatic rapid grinding instrument with a grinding power of 60 Hz. They were ground a total of 3 times, with a grinding time of 30 s and a grinding interval of 15 s after being soaked in liquid nitrogen for 10 min.

**Table 2:** HPLC gradient elution procedure.

Time (min)	A (%)	B (%)
0~30	95~90	5~10
30~70	90~78	10~22
70~85	78~65	22~35
85~95	65~20	35~80
95~100	20~20	80~80

**Table 3:** Physical properties of TDG powders with different particle sizes (*n* = 3).

Sample	TDG-CP	TDG-FP	TDG-SP	TDG-LTFP	TDG-LTSP
D_90_ (μm)	742.81 ± 24.32	379.24 ± 18.63	176.74 ± 4.77	411.87 ± 11.35	161.13 ± 6.72
D_50_ (μm)	329.61 ± 12.90	123.57 ± 7.26	16.20 ± 1.14	136.72 ± 4.75	14.36 ± 0.98
D_10_ (μm)	32.88 ± 3.59	15.42 ± 0.65	4.38 ± 0.21	16.15 ± 3.27	5.12 ± 0.33
Span	1.176	1.949	3.585	2.909	3.602
Angle of repose (°)	25.79 ± 1.03	29.25 ± 0.58	32.60 ± 0.48	29.14 ± 0.60	32.81 ± 0.69
Angle of slide (°)	32.02 ± 0.45	40.11 ± 0.50	53.01 ± 0.64	40.69 ± 0.76	53.53 ± 0.42
Bulk density (g/mL)	0.42 ± 0.01	0.47 ± 0.01	0.70 ± 0.01	0.48 ± 0.01	0.69 ± 0.01
Tap density (g/mL)	0.47 ± 0.01	0.49 ± 0.01	1.01 ± 0.01	0.48 ± 0.01	1.02 ± 0.01

## Data Availability

The original contributions presented in the study are included in the article, further inquiries can be directed to the corresponding authors.
